# MATRIX: Mental heAlth diagnostics Through Real time Intelligent unified X-AI attribution reasoning

**DOI:** 10.3389/fdgth.2025.1621271

**Published:** 2026-02-13

**Authors:** Sweety Ramnani, Kaushik Roy, Amit Sheth

**Affiliations:** Computer Science Department, Artificial Intelligence Institute, University of South Carolina, Columbia, SC, United States

**Keywords:** mental health, real-time diagnostics, attribution, explainable AI, PHQ-9, SNOMED-CT

## Abstract

Escalating prevalence of mental health issues worldwide has created an unprecedented demand for mental healthcare services, yet the shortage of qualified practitioners limits accessibility for countless individuals in need. AI has emerged as a potential solution to support mental health professionals, offering assistance that goes beyond simple diagnostic aid. This research introduces a novel AI-powered real-time diagnostic support system—MATRIX—for mental healthcare diagnostics designed to interact with users using natural language and utilizing the Patient Health Questionnaire-9 (PHQ-9), a standardized clinical tool for assessing depressive symptoms. The system classifies the interaction into a well-defined checklist and generates the most likely diagnosis through a framework termed X-AI Attribution Reasoning, which provides explainable and attributable diagnostic logic for interdisciplinary clarity. Unlike existing diagnostic support systems that primarily rely on static scoring or predefined rule sets, MATRIX integrates explainable AI (XAI) principles to deliver interpretable reasoning pathways that clinicians can trace and validate. The PHQ-9 implementation within MATRIX has been tested in controlled clinical simulations, confirming its usability and alignment with real-world assessment practices. The system not only accelerates the diagnostic process but also provides transparent explanations, detailed reasoning for such diagnoses, and clinically relevant attributions linked to standard SNOMED Concept IDs, which can be directly utilized by clinicians for documentation, referrals, and electronic health record integration while maintaining data privacy. By offering this level of insight, the system fosters a trustworthy AI-human collaboration that aids clinicians in understanding and validating each diagnostic recommendation. Interpretability within MATRIX is achieved through visual attribution maps and narrative output summaries, ensuring that decision processes remain both transparent and clinically meaningful. The integration of these features enables practitioners to focus on patient care with the assurance that AI-assisted diagnostics align with clinical standards, resulting in reduced time spent per patient and enhanced patient throughput. Our preliminary findings indicate that MATRIX achieves over 89% classification accuracy and high clinician satisfaction in pilot evaluations, demonstrating that AI-driven support systems with explainable, reasonable, and attributable real-time diagnostics can significantly enhance the capacity of mental health services and improve access to timely and effective care for those affected by mental health conditions. This study highlights the essential role of AI in enhancing both the efficiency and trustworthiness of mental health diagnostics in clinical settings, making a compelling case for the integration of AI into modern mental healthcare.

## 1 Introduction

Mental health providers face unprecedented labor shortages, with many agencies struggling to fill positions (1). According to new data released by the World Health Organization (WHO), more than 1 billion people are living with mental health disorders, with conditions such as anxiety and depression inflicting immense human and economic tolls. The global median number of mental health workers stands at 13 per 100,000 people, with extreme shortages in low- and middle-income countries (2). In the United States alone, nearly 96 unmet needs for prescribers, indicating a critical shortage of available professionals whereas in Mexico, only 1.1 psychiatrists per 100,000 population exist in the public sector, exacerbating the lack of adequate care for mental health disorders (3). This global treatment gap underscores that the shortage of mental health professionals is not limited to North America but represents a critical international public health concern. The shortage of mental healthcare providers significantly hampers the quality and accessibility of mental health services. This crisis is characterized by high vacancy rates, increased turnover, and an inadequate distribution of professionals, leading to the delayed or cancelation of patient appointments and even the closure of wards in various regions (1). A severe shortage of mental health professionals, coupled with barriers like stigma, resource constraints, and geographical limitations, creates a considerable gap in the provision of effective support.

With the rise of AI, new opportunities have emerged to transform mental healthcare, offering innovative approaches that can bridge this gap (4). AI-powered chatbots and virtual assistants represent a significant breakthrough, providing accessible, scalable, and cost-effective alternatives to traditional mental health support. It is important to distinguish, however, between AI chatbots that provide conversational support and AI-based diagnostic tools that analyze clinical data for symptom detection and prediction. While chatbots primarily facilitate self-help and engagement, diagnostic systems aim to assist clinicians in making data-driven assessments, requiring higher levels of accuracy and clinical validation. These AI tools utilize natural language processing (NLP) and machine learning (ML) to simulate therapeutic conversations, helping users engage in a more personalized and supportive experience. AI-powered diagnostic systems, on the other hand, analyze multimodal clinical data—such as patient narratives, behavioral markers, and physiological parameters—to identify symptom patterns and support clinical decision-making. AI-powered mental health diagnostics systems offer promising solutions to improve mental health diagnostics, as these systems can assist by analyzing vast amounts of data to improve predictions by differentiating between similar symptoms, aiding therapists in accurate diagnoses, and improving patient treatment outcomes (5). This capability facilitates the early detection of mental health conditions and enables timely interventions, ultimately leading to improved patient outcomes while enhancing monitoring capabilities (6).

However, the use of AI in mental health diagnostics is not without its challenges. Major challenges include clinicians’ trust, ethical concerns, and the opaque “black box” nature of AI. Explainable artificial intelligence (X-AI) addresses these issues to a certain extent by demystifying AI decision-making, allowing clinicians to understand the reasoning behind system-generated diagnoses, which helps foster trust in the technology (4). By focusing on X-AI-driven transparency, such system can not only build trust but also make mental health resources more accessible to underserved populations by providing 24/7 support and overcoming geographical barriers and stigma associated with mental health treatments (7).

Furthermore,it is noted that the validation of AI models is essential to ensure their performance aligns with real-world clinical environments, as accuracy metrics may not fully capture their effectiveness (8). The lack of standardized clinical attribution across various diagnostic models makes it more challenging to integrate with existing healthcare diagnostic systems. This research presents a novel, systematic, clinically relevant approach that unifies the power of ML with human-interpretable, AI-generated explanations and reasoning capabilities, clinically grounded through Systematized Nomenclature of Medicine—Clinical Terms (SNOMED CT) attribution. SNOMED CT provides a globally recognized, standardized vocabulary that enhances interpretability and ensures interoperability across healthcare systems, thereby facilitating the seamless exchange of clinically meaningful data. Integrating SNOMED CT (9) for structured, evidence-based attribution can further enhance diagnostic accuracy and contextual relevance. By adding a layer of clinically relevant attribution to the existing X-AI layer, the system could improve the clinical reliability of AI-assisted mental health diagnostic interventions and encourage broader acceptance within existing healthcare diagnostic systems.

## 2 Literature review

The integration of AI within healthcare systems has been widely advocated for its potential to enhance personalization, contextual adaptation, and scalability in mental health interventions (10). AI-powered chatbots and automated systems can reduce clinician workload, streamline routine processes, and extend access to mental healthcare without incurring proportional increases in cost. These benefits have positioned AI as a promising tool for broad-reaching support in mental health services. However, these advantages must be balanced against ongoing challenges including data privacy concerns, algorithmic bias, a lack of transparency, and the need for rigorous clinical validation (7, 11).

To address transparency and trust concerns, X-AI techniques such as Local Interpretable Model-Agnostic Explanations (LIME)—which approximates a complex model locally to explain individual predictions—and SHapley Additive exPlanations (SHAP)—which assigns each feature an importance value based on cooperative game theory—have been introduced (12). These tools help clinicians understand the features driving AI predictions, thereby enhancing diagnostic precision and improving resource allocation (13). Their adoption is essential in mitigating risks associated with opaque decision-making and potential misdiagnosis, ultimately supporting patient welfare (14). Techniques such as LIME and SHAP, as highlighted in recent studies, highlight the strengths (e.g., SHAP’s consistency) and limitations (e.g., LIME’s instability), emphasizing the need for careful selection of explanation methods depending on the clinical context. As AI systems become increasingly pervasive in mental healthcare, ethical and regulatory considerations gain critical importance (Algorithm). Clinical validation ensures that AI-driven diagnostics meet standards of transparency, safety, and accuracy, while proper clinical attribution enhances trust between clinicians and patients (11). Attribution methods—used to assess feature importance and understand model decisions—are crucial for mitigating algorithmic bias and ensuring equitable care for diverse populations (15). Clear attribution frameworks also strengthen the ethical foundation of AI-based mental health diagnostics by improving transparency and accountability (16, 17). SNOMED CT plays an increasingly central role in advancing the reliability, interoperability, and clinical relevance of AI systems. As a comprehensive, standardized clinical terminology, SNOMED CT enhances the alignment of AI-generated insights with globally recognized medical standards, improving both accuracy and trust (18). The linkage between SNOMED CT and model outputs can occur through both *post hoc* mapping, where predicted diagnostic terms are translated into SNOMED concepts, and integration during model training, where structured terminology is embedded directly into the model to guide representation learning and terminology-aware predictions. This dual integration pathway enhances the interpretability and utility of explanation tools such as LIME and SHAP. Recent empirical studies demonstrate that embedding SNOMED CT into AI pipelines improves fairness by reducing terminology-related bias and enhances diagnostic consistency across healthcare settings (19). Case studies have shown that SNOMED CT-driven models yield improved clinical decision support outcomes, particularly in mental health contexts where linguistic ambiguity often complicates diagnosis. Furthermore, the structured representation of patient symptoms supports personalized and context-specific treatment planning while enabling scalable, interoperable, and clinically meaningful AI applications. Thus, SNOMED CT-based clinical attribution provides a robust foundation for trustworthy X-AI, addressing key challenges related to transparency, fairness, and ethical compliance in mental health diagnostics.

## 3 Demystifying PHQ-9 and SNOMED CT

### 3.1 Patient health questionnaire

**Figure 1:**
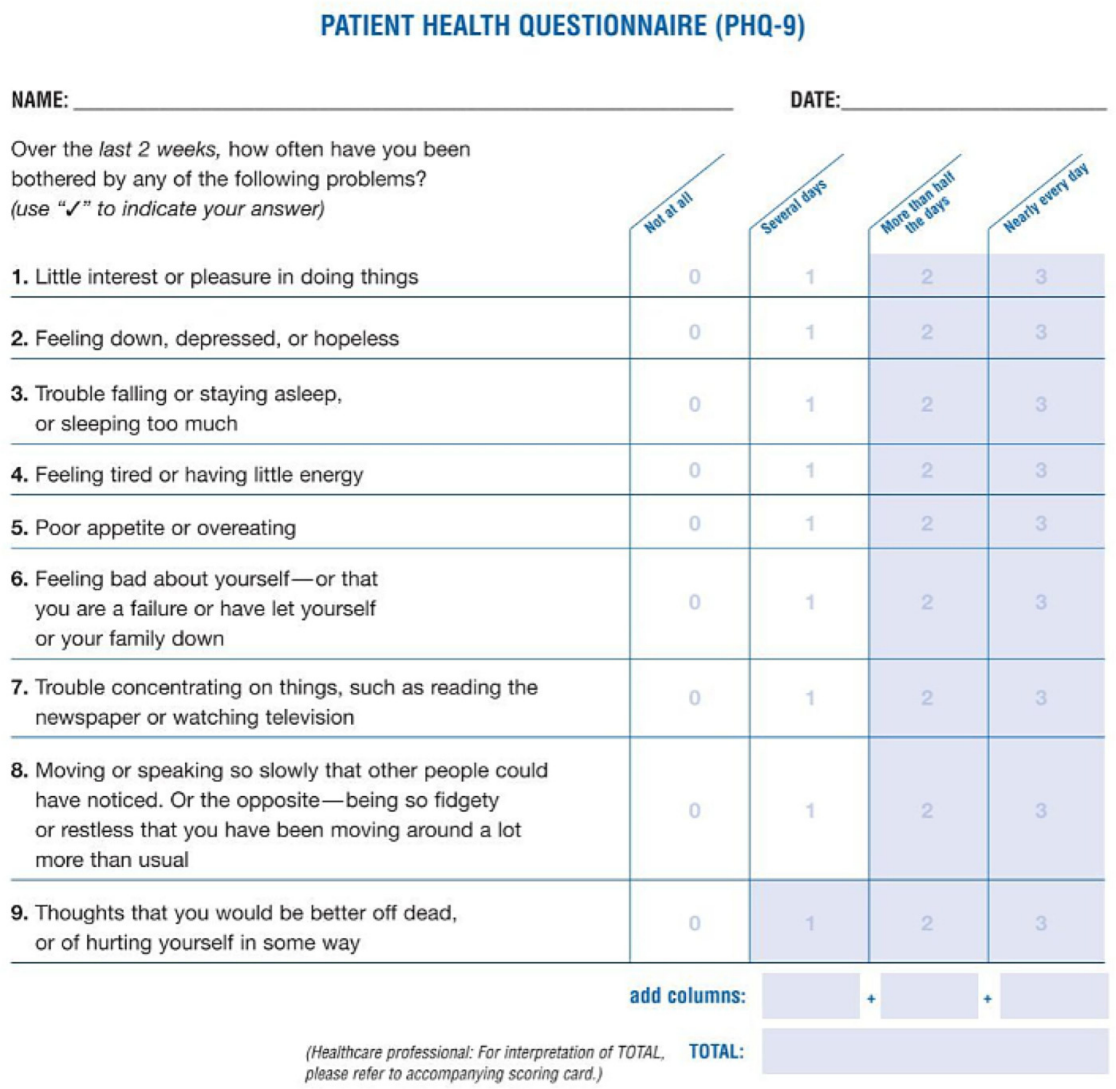
Standardized PHQ-9 checklist. Reproduced from “Patient Health Questionnaire (PHQ) Screeners” by Drs. Robert L. Spitzer, Janet B.W. Williams, Kurt Kroenke and colleagues.

PHQ-9 is a widely used, standardized self-administered tool for assessing the severity of depression. It consists of nine questions based on the diagnostic criteria for major depressive disorder outlined in the Diagnostic and Statistical Manual of Mental Disorders (DSM) (20). Each question asks the respondent to rate the frequency of specific depressive symptoms over the last 2 weeks on a scale from 0 (not at all) to 3 (nearly every day). PHQ-9 scores range from 0 to 27, with higher scores indicating greater severity of depression. The major categories are as follows: 0–4, minimal depression; 5–9, mild depression; 10–14, moderate depression; 15–19, moderately severe depression; and 20–27, severe depression. The tool is commonly used in clinical settings to screen for depression, monitor treatment progress, inform initial diagnostic decisions. The standardized format is shown in Figure 1.

### 3.2 SNOMED CT

SNOMED CT is the world’s most comprehensive, multilingual clinical terminology system (21). It contains over 300,000 concepts with associated terms, synonyms, attributes, and hierarchical relationships. SNOMED CT is a designated U.S. standard for electronic health information exchange and is essential for accurately capturing patient problems, medical histories, and clinical documentation in electronic health records (EHRs).

**Figure 2:**
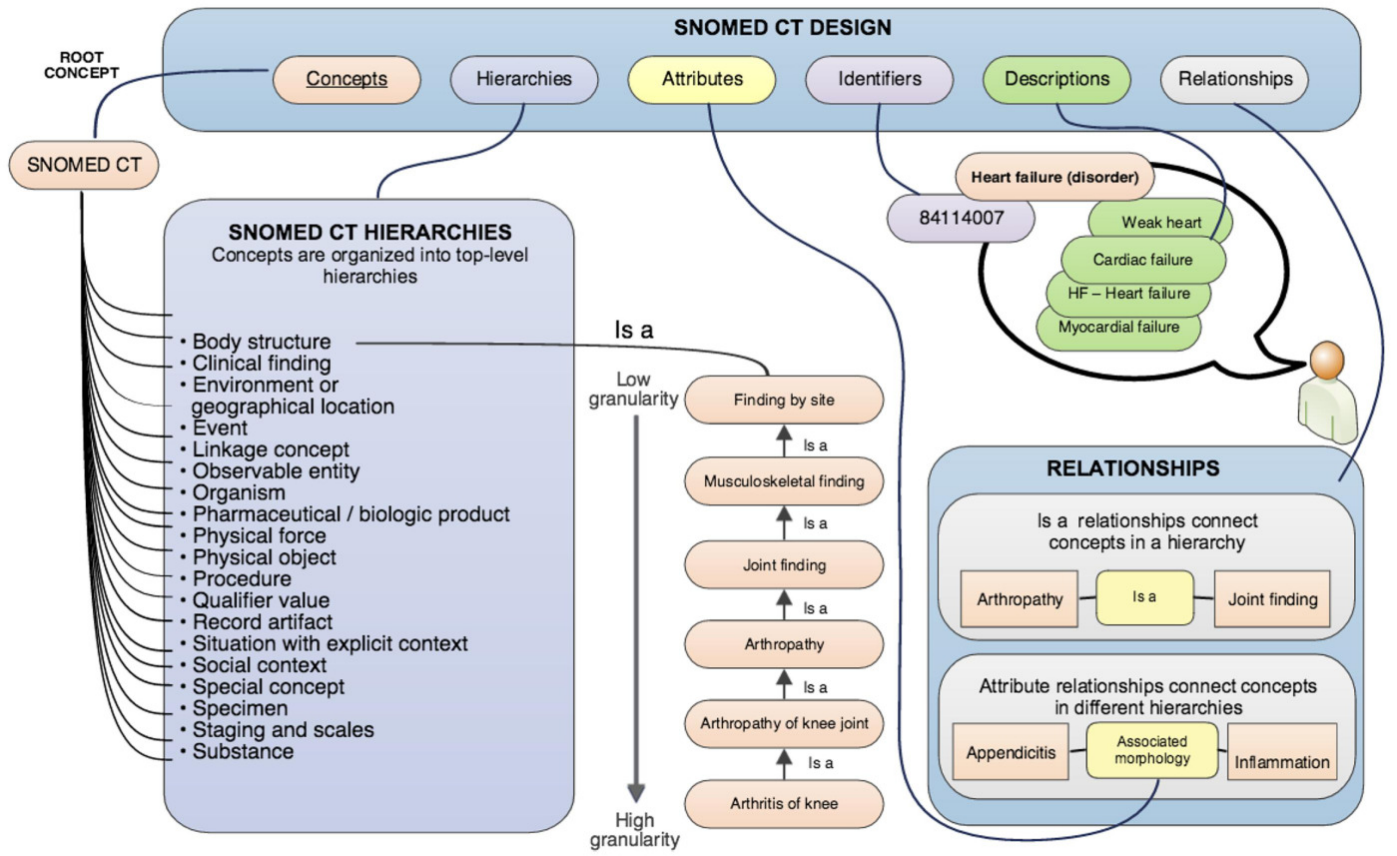
Standardized SNOMED CT.

SNOMED CT supports semantic interoperability by standardizing how clinical information is represented and exchanged across healthcare systems. Each concept in SNOMED CT (9) corresponds to a unique clinical meaning and is referenced using a machine-readable numeric concept identifier that is not inherently interpretable by clinicians. For example, the concept “Depressed mood (finding)” is represented by the concept ID 366979004, providing an unambiguous reference for documentation, analysis, and integration into computational systems. Figure 2 illustrates the hierarchical structure through which SNOMED CT organizes clinical concepts into expressive and computable relationships. Together, PHQ-9 and SNOMED CT provide the clinical foundation of our system: PHQ-9 supplies structured symptom data, while SNOMED CT ensures standardized terminology and semantic consistency for diagnostic interpretation.

## 4 Our approach

**Figure 3:**
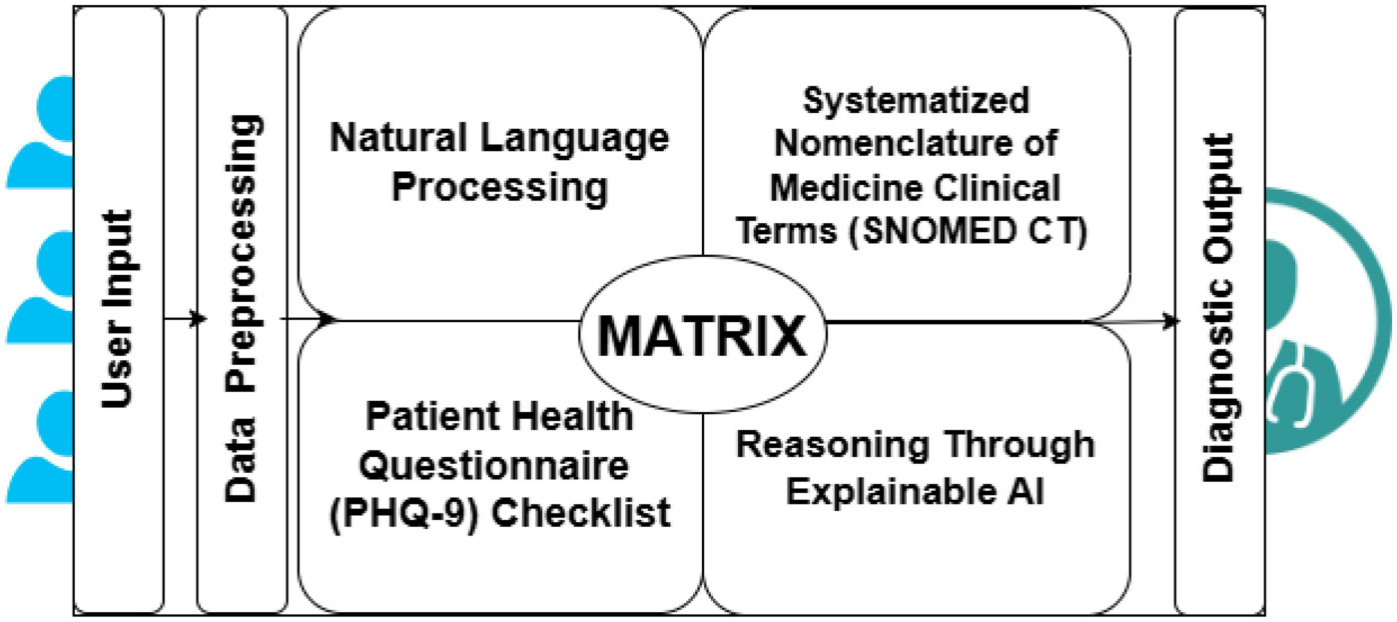
MATRIX framework.

The MATRIX framework is depicted in Figure 3. The algorithm underlying this tool leverages NLP and ML techniques to detect mental and health symptoms based on conversational responses with the respondent in real time. The algorithmic steps and specifications of various data resources are detailed in the following sections. User responses are parsed in real time through an NLP pipeline that identifies significant linguistic markers, extracting key features such as emotional cue indicators and patterns, which are then analyzed using the PHQ-9 SYMPTOM ONTOLOGY dataset (detailed in the next section) to enhance the precision of PHQ-9 questionnaire classification.

Users’ current state of mind shared in a natural language format, parsed through an NLP pipeline reaches the next stage where it is further analyzed—guided by questions rooted in the standarized PHQ-9 questionnaire, screening depressive symptoms and assessing symptom severity(as per major categories described in the previous section) based on the input received from the NLP pipeline.

This severity score guides the rule-based internal system in generating the most likely diagnosis. To achieve an accurate diagnosis based on symptom severity scores, each depressive symptom feature is weighted and assessed by a five-layered (obtained through hyperparameter testing) neural network model trained on a vast dataset of the PRIMATE ontology, which contains widely annotated datasets. This system-generated diagnosis is then fed to a reasoning layer, which draws on clinical guidelines to understand why specific symptoms have been flagged and generates explanations with the use of language model, BERT model in this case, to ensure privacy of users’ data (more details are provided in the Results section), enhancing the system’s interpretability and helping clinicians understand better the rationale behind specific diagnosis.

Further refining its diagnostic utility, our system attributes detected symptoms and diagnoses by fetching relevant SNOMED CT IDs in real time, which are recognized clinical codes used in EHRs worldwide. By dynamically associating symptoms with SNOMED CT identifiers, the algorithm ensures that each AI-based classification and diagnosis aligns with clinically accepted mental health diagnoses, providing a direct pathway from initial symptom detection to actionable diagnostic insights.

**Figure 4:**
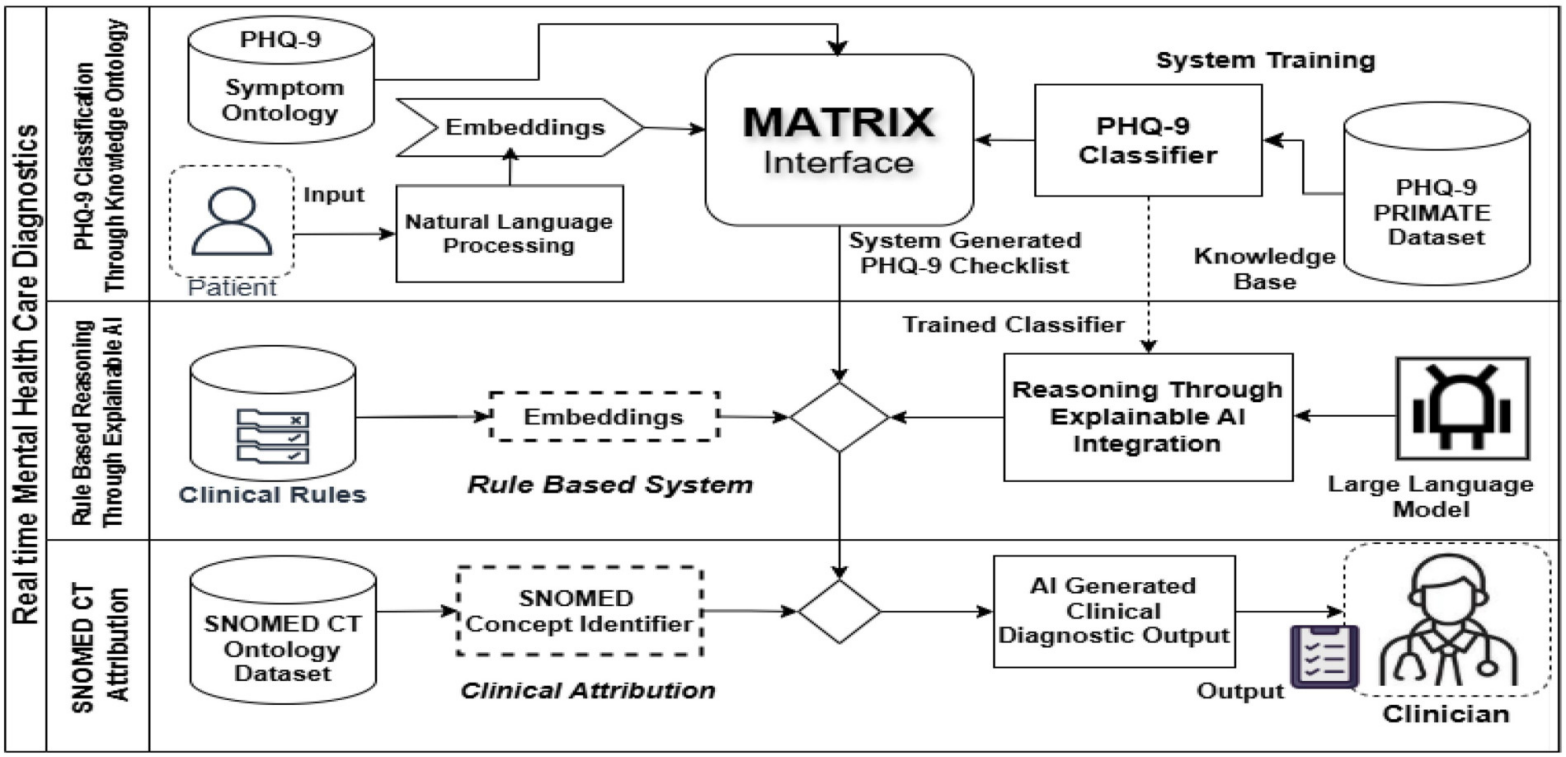
MATRIX flowchart.

The MATRIX model (flowchart depicted in Figure 4) dynamically evaluates user input in real time, mapping it onto a spectrum of symptom severity, generating the most likely diagnosis, and embedding a layer of reasoning that offers transparent explanations for the system’s decision and attributing the diagnosis with a specific clinically relevant SNOMED concept identifier. Mental health practitioners can thus access these AI-generated recommendations, along with clinically relevant justifications, all in real time, enabling them to review, adjust, or supplement the AI suggestions seamlessly.

## 5 Data resources

### 5.1 PRIMATE dataset

This dataset consists of 2,000 publicly available Reddit posts, each describing users’ mental health experiences and containing binary annotations (“yes”/“no”) indicating whether the text reflects PHQ-9 symptoms (S1–S9). The dataset predominantly includes posts from English-speaking users aged 18–35 years, based on platform demographics reported in prior studies, although exact demographic identifiers are not included to maintain anonymity. As the dataset was sourced from existing public-domain Reddit posts, no additional informed consent was required; the original study complied with Reddit’s terms of service, and ethical approval was obtained at the dataset creation stage (22). Annotations were contributed by five crowd-workers, with an interannotator agreement of 67%. To strengthen reliability, mental health professionals conducted a secondary evaluation, reaching an agreement of 85%. In addition, the original dataset reports Cohen’s = 0.71 (95% CI: 0.68–0.74), indicating substantial interannotator agreement and reinforcing the quality of labeled PHQ-9 symptom data.

### 5.2 PHQ-9 symptom ontology

This ontology contains more than 500 dictionary words categorized based on similarity and semantic closeness to symptoms corresponding to each of the nine standard PHQ-9 questions (20). The ontology was compiled through a hybrid approach: expert consensus from licensed psychologists who reviewed symptom clusters, lexicon-based expansion using standard psychological terminology, and NLP-assisted validation, in which embeddings were used to verify semantic proximity between terms. This multistage construction process ensures clinical coherence and strong alignment between textual symptom cues and the nine PHQ-9 diagnostic categories. For illustration, the term “loss of interest” is mapped to PHQ-9 item 1 through similarity scoring and expert validation.

### 5.3 SNOMED CT database

The SNOMED CT lexicon includes over 50,000 clinical terms and their associated concept identifiers, as maintained by SNOMED International (UK). Each entry contains a unique concept ID and a case-significance flag for standardized usage across EHR systems (9). In this system, the SNOMED CT database is accessed locally rather than via API to avoid transmitting clinical text externally. This approach minimizes privacy risks and improves performance during real-time inference. “Closest match” retrieval is performed using a semantic similarity algorithm based on cosine similarity between term embeddings, supplemented with rule-based filters to ensure medically valid matches. For example, when the model identifies “moderate depression,” the reasoning output links PHQ-9 items (e.g., elevated scores in S1, S2, and S6) to the SNOMED concept “Moderate major depression (ID: 370143000),” along with an explanation of the contributing symptoms. Clinicians retain full authority to edit or override any system-generated SNOMED CT mappings; their feedback is logged and incorporated into subsequent fine-tuning cycles, supporting continuous improvement and clinical safety.

## 6 Methodology

**Algorithm:** 

Initial system training: The system begins with initial training on the PHQ-9 PRIMATE dataset. It learns to identify relevant features from annotated data. Once trained, the system can create the PHQ-9 checklist by itself from user input. NLP preprocessing component: Tokenization, stop word removal, and feature extraction techniques are applied to the raw input to create embeddings, which are further processed at the next stage. Embedding component: Embeddings are dense, low-dimensional vector representations of words, sentences, and documents. These vectors capture semantic meanings and relationships between words or phrases. This component creates embeddings for input tokens. It is done basically to contextualize symptoms based on the user’s input responses. It extracts key symptom indicators and sentiments that correlate with depression criteria. PHQ9 checklist generation: The MATRIX system, pre-trained in step 1 to generate the PHQ-9 checklist, processes each question iteratively and determines the answer as “Yes” (if the symptom is present) or “No” (if the symptom is absent) based on the embeddings input. It leverages the PHQ-9 symptom ontology for reference. PHQ-9 scoring module: In the next stage, embeddings for the clinical rules are created. The system takes those embeddings, along with the system-generated PHQ-9 checklist from the previous stage, and calculates the PHQ-9 severity score to determine depression severity. At this stage, the score is used to calculate diagnostic thresholds (e.g., mild, moderate, moderately severe, severe depression), and an appropriate diagnosis is indicated. Reasoning component: After the system provides a diagnosis and a checklist, the capability of large language models is leveraged to provide the reasoning and generate explanations in natural language for clinicians’ aid. Diagnostic clinical attribution: This stage maps the system-generated diagnosis with SNOMED Concept IDs to ensure standardization in clinical terminology, providing clinicians’ with clear attributions for each diagnosis. It refers to the SNOMED CT database to find the closest match and fetches the concept identifier in real time. Explainability (X-AI) layer: It incorporates human-readable explanations (from the previous layer) of diagnostic results. It includes the symptoms and responses that contributed to the overall PHQ-9 severity score, integrating *highlighted* symptoms and relevant SNOMED IDs, to enhance transparency and trust in system-generated diagnoses. Diagnosis output: The system creates a completes clinical diagnosis incorporating the PHQ-9 checklist, symptoms severity score, severity level, and reasoning through proper explanations and attribution with an appropriate SNOMED CT identifier in an intuitive format.

MATRIX, as a comprehensive unified interface, sets a new standard in digital mental health support by enabling rapid assessments and AI-driven responses. By streamlining the initial evaluation process, it reduces consultation time, allowing mental health professionals to concentrate on critical interventions. In addition, this approach significantly enhances accessibility, extending support to underserved populations through digital platforms. It empowers practitioners with reliable, data-driven insights for well-informed decision-making. By automating diagnostic documentation and structuring responses for a quick review, the system optimizes the efficiency of mental healthcare services, improving patient throughput and saving valuable clinician time.

## 7 System technical specification

The system integrates components for data preprocessing, feature extraction using transformers, and classification using a custom-built neural network. The system accepts input from a JSON file containing textual posts (post_text) for initial system training and the PHQ-9 symptom ontology file, with columns representing PHQ-9 questions and their associated symptoms. The data preprocessing pipeline iterates through the posts, identifying relevant symptoms from the ontology, and associates them with corresponding PHQ-9 questions. Feature extraction and embedding generation: Text embeddings are generated using the Sentence Transformers (all-MiniLM-L6-v2) for semantic representation. Input text is tokenized and padded to 128 tokens, and mean pooling is applied over token embeddings to produce a fixed-size vector representation.

Further, a feedforward neural network is employed for multiclass classification. The architecture comprises an input layer that matches the dimensionality of the embedding vectors, a fully connected hidden layer with 128 neurons and ReLU activation, and an output layer that provides a probability distribution across PHQ-9 questions using the Softmax activation function. For classification decisions, a confidence threshold of 0.55 is applied. Predictions with probabilities below this threshold are suppressed to reduce false positives; otherwise, raw probabilities contribute to downstream reasoning. The network is optimized using the Adam optimizer with a learning rate of 0.001 (obtained through hyperparameter testing), and the loss is calculated using the CrossEntropyLoss function. The model is trained for 32 epochs, optimizing weights through backpropagation. The evaluation is performed by comparing predicted classifications with true labels for the held-out test set to assess performance. Moreover, to generate a diagnosis, the system counts the occurrences of predicted PHQ-9 questions and identifies patterns that indicate major depressive symptoms. The diagnostic results summarize the predominant questions and their frequency, providing insights into symptom distribution. The model generates predictions in the form of a system-generated PHQ-9 checklist and most likely diagnosis.

**Figure 5:**
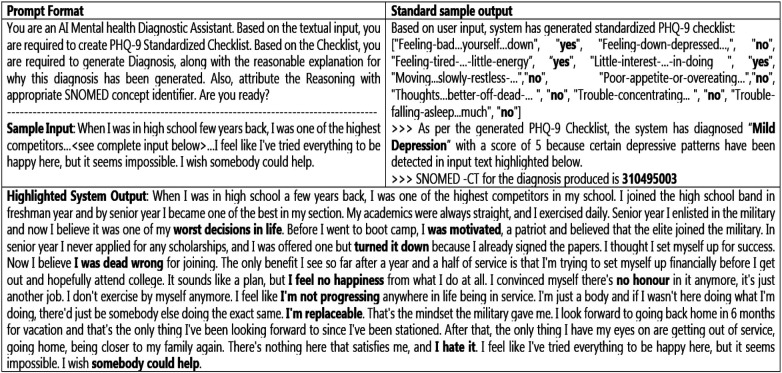
System input, output, and prompt formats.

For the next layer of reasoning, the system is designed to leverage a pretrained PyTorch-based model to analyze input from the previous layer and produce diagnostic explanations, complete with system evaluation metrics. The system processes the inputs through a trained model (described above) and tokenizer. Open-source model BERT-base-uncased, a standard 110M-parameter pretrained model, is utilized, which provides textual reasoning aligned with observed symptoms in the previous layer. For each question in the checklist, the system offers explanations, linking and highlighting the detected patterns (cues) to the corresponding PHQ-9 criteria. Diagnostic reasoning is contextualized, ensuring that the level of depression aligns with PHQ-9 standards and observed symptom patterns, which diminishes the occurrence of hallucinations. Sample system input, output, and prompt formats are presented in Figure 5.

The attribution layer matches and attributes system diagnoses against SNOMED CT terminology in real time using both regular expression filtering and semantic embeddings. The system is designed to enhance the precision of diagnosis-to-concept mapping by leveraging Sentence Transformers for semantic similarity calculations. The input from the previous layers is tokenized and converted to lowercase to improve matching accuracy. The diagnosis is divided into keywords, and a regex pattern is constructed. The “term” column in the SNOMED dataset is filtered for matches containing any of the keywords, ensuring broader term coverage. If multiple terms match, the system uses Sentence Transformers to compute embeddings for the matched terms and the diagnosis. Cosine similarity scores are calculated to identify the term most semantically similar to the diagnosis input. Once the closest match is determined (presented as a similarity score), the system extracts and returns relevant information, including “moduleId,” “conceptId,” “languageCode,” and the matched SNOMED concept. A detailed message is included in the output to enhance interpretability depicted in Table 1.

**Table 1:** System diagnosis information for the attribution layer.

SNOMED	Diagnosis input: major depression	
CT detail	Field	Sample values
1	ModuleId	900,000,000,000,207,008
2	ConceptId	123,456,789
3	LanguageCode	en
4	TypeId	900,000,000,000,003,001
5	CaseSignificanceId	900,000,000,000,448,009
6	Snomed_concept	Major depression
7	Similarity score	0.91
8	*Message	This is system-generated diagnosis. More information can be found in the following link: https://www.snomed.org/get-snomed

System testing is carried out on a subset (44 samples) of manually validated diagnosis-to-SNOMED mappings. The combination of regex filtering and semantic embeddings ensures improved accuracy. We believe that with the proposed enhancements, the system can become a highly reliable tool for mapping clinical data. To support clinical deployment, the system reports the following: average inference time per input, 38 ms on an NVIDIA T4 GPU; end-to-end latency (preprocessing → reasoning), 112 ms; and peak memory usage during inference, 1.3 GB. The code is made available as open source on https://github.com/Sweenderella/MATRIX for ease of reproducibility.

## 8 Evaluation results and discussion

### 8.1 Ground data generation and interpretation of metrics

Annotated instances from the PRIMATE dataset was used as ground data. The MATRIX system was rigorously tested against these ground datasets along with several state-of-the-art models, such as Generative Pretrained Transformer 3 model with 175B parameters, Gemini with approximately 3̃00B parameters, and Claude with approximately 70–100B parameters. The system was given a standard initial prompt that clearly defined the task. A system-generated checklist for the standard questionnaire was recorded for every model under testing, and a confusion matrix was created. Performance was measured using standard evaluation metrics (refer Equations 1–4), where TP refers to true positive values, TN refers to true negative, and FP and FN refer to false positive and false negative values, respectively.

$$\text{Accuracy}=\frac{\mathrm{TP}+\mathrm{TN}}{\mathrm{TP}+\mathrm{TN}+\mathrm{FP}+\mathrm{FN}}$$ (1)

Accuracy measures the proportion of correctly classified instances out of the total number of instances.

$$\text{Precision}=\frac{\mathrm{TP}}{\mathrm{TP}+\mathrm{FP}}$$ (2)

Precision quantifies the proportion of true positive predictions among all positive predictions, reflecting reliability.

$$\text{Recall}=\frac{\mathrm{TP}}{\mathrm{TP}+\mathrm{FN}}$$ (3)

Recall measures the proportion of true positive instances that were correctly identified out of all actual positives.

$$F1=2\cdot \frac{\text{Precision}\cdot \text{Recall}}{\text{Precision}+\text{Recall}}$$ (4)

The F1 score is the harmonic mean of precision and recall, providing a balanced measure of the performance of a model, especially in imbalanced datasets. These findings are presented in Table 2.

**Table 2:** Performance metrics for PHQ-9 checklist generation.

Model	Accuracy	Precision	Recall	F1 score
**MATRIX**	**0.89**	**0.87**	**0.91**	**0.89**
GPT-3	0.88	0.87	0.89	0.88
Gemini	0.85	0.84	0.86	0.85
Claude	0.86	0.79	0.82	0.80

Bold values indicate improved PHQ-9 checklist generation performance, indicating greater predictive accuracy, balanced precision and recall, and more reliable identification of clinically relevant depressive symptoms compared to baseline models.

The proposed model achieved an accuracy of 89%, indicating its effectiveness in correctly classifying PHQ-9 checklist responses compared to a few other benchmark models. A precision score of 87% reflects the ability of the model to reliably generate positive responses, minimizing false positives. With a recall score of 91%, the model effectively identifies all relevant instances, reducing false negatives in checklist generation. The F1 score of 89% demonstrates a balanced tradeoff between precision and recall, underscoring the robustness of the model for PHQ-9 checklist generation. The results confirm that the proposed model shows improvements against traditional benchmarks after targeting training with the PRIMATE dataset, particularly in recall and F1 score, underscoring its ability to accurately assess depression severity from input and align symptoms with clinically validated PHQ-9 standards.

### 8.2 Context reasoning understanding and evaluation metrics

To further assess the ability of the system to generate explanations and provide reasoning, we evaluated the performance of multiple pretrained models in our proposed model, including DistilBERT (23), BERT-Base (24), RoBERTa-Base and RoBERTa-Large (25), and DeBERTa-Base and DeBERTa-Large (26).

Despite the impressive performance of large language models (LLMs) like GPT-3 and Gemini in tasks involving complex reasoning, we chose not to use them other than testing our proposed models with ground data in the previous section due to significant concerns regarding data privacy. LLMs require cloud-based processing, which can inadvertently expose sensitive user data, as the input is sent to external servers for processing. Given that our application, MATRIX, is designed as a standalone system that processes data locally, using LLMs would compromise the privacy and security of user information. In contrast, BERT-based models operate entirely within the local environment, ensuring that no data leaves the user’s machine, thus maintaining strict privacy standards. Moreover, although LLMs exhibit exceptional reasoning capabilities, the BERT family of models has been shown to deliver highly accurate results in context understanding and reasoning tasks, while addressing the data privacy issues that are critical in healthcare and other sensitive domains. Thus, BERT-based models strike an optimal balance between performance and user data protection, making them a more suitable choice for our application.

To assess the contextual understanding and reasoning capabilities of our proposed model, we employed several advanced metrics, including cosine similarity, semantic similarity, and contextual relevance scores. These metrics allow us to quantify how well the model captures and responds to the contextual meaning of the input data.

*Cosine similarity* measures the similarity between two non-zero vectors by computing the cosine of the angle between them. It is commonly used to assess how closely related two texts are in terms of their semantic meaning. The formula for cosine similarity is given as follows:
  $$\text{Cosine similarity}=\frac{A\cdot B}{\left\|A\|\|B\right\|}$$
  where
  A and B are the embedding vectors of the input context and generated response, respectively.
  A⋅B is the dot product of the two vectors.
  ‖A‖ and ‖B‖ are the magnitudes (Euclidean norms) of vectors A and B, respectively.
  A cosine similarity score close to 1 indicates high semantic similarity, implying that the generated response is highly aligned with the input context, while a score closer to 0 suggests weak alignment.
The *Semantic similarity score* measures the degree to which two sentences or pieces of text convey the same meaning. Using advanced embedding models, such as Sentence-BERT or Universal Sentence Encoder, we computed the similarity between the semantic representations of the input context and the generated response. The similarity score is derived by comparing the cosine of the angle between the two sentence embeddings.
  $$\text{Semantic similarity score}=\cos(\theta )=\frac{A\cdot B}{\left\|A\|\|B\right\|}$$
  where A and B represent the semantic embeddings of the input and generated response, respectively. Higher values of semantic similarity indicate that the model has captured the contextual meaning and provided a more coherent and relevant explanation.
The *Contextual relevance score* (*CRS*) evaluates the extent to which the generated explanation maintains logical consistency with the input context. This score is computed using contextual embedding model BERT, which assesses how well the explanation fits within the broader input context as per the following metric:
  $$\text{Context relevance}=\frac{\text{Relevant contextual match}}{\text{Total contextual potential}}$$
  A higher context relevance score reflects that the model’s response is more relevant and logically consistent with the input context.

Given the importance of these metrics in evaluating the reasoning ability of the proposed system, we incorporated these scores into our evaluation framework. The results of this evaluation are detailed in Table 3. It is worth noting that we chose BERT-based models over LLMs due to privacy concerns. As MATRIX is a standalone application, processing data locally ensures that user data are not transmitted to external servers. This design decision guarantees privacy while still maintaining high performance in context understanding and reasoning tasks.

**Table 3:** Context reasoning evaluation for MATRIX.

Model (parameters)	Cosine similarity	Semantic similarity score	Context relevance score
DistilBERT (66M)	0.85	0.80	0.75
DeBERTa-B (86M)	0.92	0.91	0.90
BERT-B (110M)	0.90	0.88	0.85
RoBERTa-B (125M)	0.91	0.89	0.88
DeBERTa-L (304M)	**0.93**	**0.92**	**0.91**
RoBERTa-L (355M)	0.92	0.90	0.89

Bold values indicate stronger contextual reasoning performance, reflecting greater semantic alignment, improved contextual relevance, and more accurate representation of meaning through DeBERTa-L in the MATRIX framework.

The results presented highlight the strong performance of these models in maintaining semantic alignment and contextual coherence across a diverse range of inputs. DeBERTa-L and DeBERTa-B, in particular, achieved the highest scores across all metrics, demonstrating their ability to generate contextually relevant and semantically consistent responses.

Offering clinicians an efficient, transparent, and clinically aligned diagnostic tool while maintaining users’ privacy allows mental health professionals to serve more patients with a higher degree of accuracy and confidence. This scalability is crucial amid the rising demand for mental health services and the shortage of qualified practitioners. Furthermore, by automating key aspects of the diagnostic process, the system frees clinicians to focus on in-depth patient care, potentially improving patient outcomes and satisfaction. Its alignment with clinical standards and emphasis on transparency also make it a trustworthy and adaptable tool, suitable for diverse healthcare settings where reliable, AI-assisted diagnostics are urgently needed.

It is worth noting that we are currently engaged in a thorough improvement of the quality of outcomes, consulting mental healthcare domain experts to obtain a comprehensive report on human agreement scores. This study represents an encouraging preliminary exploration of the real-time standalone MATRIX system in the form of a mental health companion, offering real-time clinically relevant assessments.

## 9 Conclusion

The implemented system provides a comprehensive framework for generating, classifying, and explaining PHQ-9 checklists. By leveraging advanced language models such as DeBERTa and RoBERTa, the system demonstrated state-of-the-art performance in aligning AI-generated outputs with established clinical standards. Rigorous evaluations using the PRIMATE dataset highlighted its ability to generate accurate, reliable, and interpretable diagnostics, making it a transformative tool in mental healthcare. The integration of standardized assessments, such as PHQ-9, ensures diagnostic consistency, while mapping AI-driven reasoning to real-time SNOMED CT concepts fosters clinical relevance and actionable insights. This capability not only reduces diagnostic time but also enhances trust of clinicians in AI-driven recommendations, ultimately supporting more efficient workflows and improved patient outcomes.

By addressing critical challenges in mental health diagnostics, the system exemplifies the potential of X-AI to bridge the gap between technical innovation and clinical application while maintaining the privacy of users’ data. Its scalable and accessible design positions it as a valuable resource in addressing the growing demand for mental health services, particularly in underresourced and geographically dispersed populations. Moreover, the novel MATRIX interface enables real-time interaction and reasoning, enhancing both patient engagement and clinician efficiency. The study’s emphasis on transparency, accuracy, and semantic reasoning ensures that the system not only delivers clinically reliable outputs but also supports practitioners in delivering high-quality care in diverse healthcare environments.

## Data availability statement

The datasets presented in this study can be found in online repositories. The names of the repository/repositories and accession number(s) can be found in the article/Supplementary Material.
